# The mRNA expression of IGF-1 and IGF-1R in human breast cancer: association with clinico-pathological parameters

**DOI:** 10.1186/1477-3163-5-16

**Published:** 2006-05-25

**Authors:** W Al Sarakbi, YM Chong, SLJ Williams, AK Sharma, K Mokbel

**Affiliations:** 1 Breast Unit St.George's Hospital and Medical School Tooting, London SW17 0QT, UK

## Background

The insulin-like growth factor-I (IGF-I) receptor is a member of a large family of transmembrane signal transducing molecules. The IGF system is composed of IGF ligands, receptors, and binding proteins. These system components form a highly regulated network of interactions both among themselves and between other biologic signalling pathways [1,2].

There are two characterised ligands, IGF-I and IGF-II whose activities are mainly mediated by type I and II receptors [1,3]. IGF-1R is necessary for the normal progression through the cell cycle and for normal growth and development [4,5].

The principal pathways for transduction of the IGF signal are mitogen-activated protein kinase and phosphatidylinositol 3'-kinase [6,7]. After ligand-dependent receptor autophosphorylation, the IGF-IR phosphorylates a series of adaptor proteins, including insulin receptor substrate-1, to activate intracellular signalling cascades [7]. The protein kinase pathway is responsible for the mitogenic signal observed after IGF stimulation but may also be accountable for cell survival in cells over expressing the IGF-1R [8]. The IGF system has been implicated in promoting mitogenic, metastatic, and antiapoptotic phenotypes in breast cancer. As a consequence of the ability of IGF to promote tumorigenesis, pharmacologic interventions targeting the IGF system are being devised [9-11]. It is believed that IGF-1 can affect breast cells through endocrine, autocrine, and paracrine mechanisms [12-14].

We have recently reported using RT-PCR that IGF-1 is lower in breast cancer tissue than in adjacent non-cancerous breast tissue (ANCT) thus supporting the paracrine hypothesis [15]. The same study demonstrated no significant difference in the expression of IGF-1R between breast cancer and ANCT, neither was there any significant correlation between PCR and immunohistochemistry (IHC) expressions of IGF-1R [15].

This aim of our study is to evaluate the relationship between mRNA expression levels of IGF-1 and various clinicopathological parameters.

## Methods

### Specimens

A total of 31 patients (*n *= 31) with diagnosis of operable breast cancer were randomly selected. Local protocols were followed regarding ethical approval and patients' consent. A specimen of tumour and another specimen of ANCT were obtained from each patient. They were frozen in liquid nitrogen within 30 minutes of excision and stored at -70°C until use.

### RNA extraction

Total cellular RNA was extracted from cancerous tissue and ANCT using Total RNA Isolation Reagent (Advanced Biotechnologies) according to manufacturer's protocol. Approximately 10 mg of cancerous tissue was homogenized. A larger amount of ANCT (20 – 50 mg) was used as high fat content made it difficult to obtain a sufficient RNA concentration for analysis. Quantification of RNA following treatment with DNase was carried out in triplicate using Ribogreen reagent (Molecular Probes Europe BV, Leiden, Netherlands) according to manufacturer's protocol.

### RT-PCR

Taqman RT-PCR was carried out on each sample using the SYBR Green PCR Mastermix (Applied Biosystems, Warrington, UK) and the ABI PRISM 7700 Sequence Detector (Perkin Elmer Applied Biosystems, Warrington, UK) according to manufacturer's protocol. The RNA extraction and the preparation of the RT-PCR mix were carried out in different rooms. Preparation of the RT-PCR mix was carried out in an extraction hood.

The sequences for the forward and reverse oligonucleotide primers and probes were designed using Primer Express Software (PE-Applied Biosystems, Warrington, UK) and were intron spanned to prevent amplification of genomic DNA. The oligonucleotide sequences of these primers and probes are given in table 1.

**Table 1:** Sequence of Taqman primers and probes for RT-PCR

**Name of gene**	**Forward Primers**	**Reverse Primers**	**Probe**
IGF-1	5-CTT CAG TTC GTG TGT GGA GAC AG-3	5-CGC CCT CCG ACT GCT G-3	5-TTT TAT TTC AAC AAG CCC ACA GGT ATG GC-3
IGF-1R	5-CTC CTG TTT CTC TCC GCC G-3	5-ATA GTC GTT GCG GAT GTC GAT-3	5-TGG CCC GCA GAT TTC TCC ACT CGT-3

The conditions for the reverse transcription were 50°C for 2 min, 60°C for 10 min and 92°C for 5 min. The polymerase chain reaction step was carried out for 50 cycles for 20 sec at 92°C and 25 sec at 62°C. For negative controls, RNase-free water was used in the RT-PCR instead of RNA. For positive controls, 18S ribosomal mRNA was used. The relative levels of IGF-1 and IGF-1 R mRNA expression were calculated by comparing their readings (arbitrary units) to the readings of a housekeeping gene (18S ribosomal mRNA). The association between the relative levels of IGF-1 and IGF-1R mRNA and clinicopathological parameters was examined using statistical analysis of variance. The clinicopathological parameters included patients' age, tumour size, grade vascular invasion, estrogen receptor (ER) status, lymph node status, and associated DCIS.

## Results

All 31 patients had their operations performed in the period between January 1997 to December 1998 and underwent either a mastectomy or a wide local excision together with lymph node clearance or sampling. The median age was 69 and 7 patients were below the age of 50. Six patients were pre-menopausal. All samples contained invasive ductal carcinoma and 14 of these also contained ductal carcinoma in-situ (DCIS). 18 cases were positive for estrogen receptor (ER) immunostaining. Histological grading of cancerous tissue revealed 3 cases of grade 1, 10 cases of grade 2 and 17 cases of grade 3. The histology grade of one case could not be determined.

All 31 specimens had detectable levels of mRNA for both IGF-1 and IGF-1R. The median values of relative expression of IGF-1 mRNA were 0.469 (0.281–0.711) 0.762 (0.338–0.874) in tumour and ANCT respectively (p = 0.0002).

The IGF-1 receptor values were 0.118 (0.0763–0.1685) in tumour specimens and 0.0944 (0.0356–0.1242) in ANCT. The difference was not statistically significant indicating a more universal expression.

We observed a significant association between IGF-1 mRNA expression and lymph node status (0, 1–3, >3 positive lymph nodes, p = 0.0032).

There was no significant association between IGF-I mRNA expression and patients' age (p = 0.3683), tumour size (p = 0.855), grade (p = 0.8789), vascular invasion (p = 0.5717), ER status (p = 0.5498) or the presence of DCIS (p = 0.8456).

There was no significant association between IGF-1R mRNA expression and patients' age (p = 0.115), tumour size (p = 0.7417), grade (p = 0.1084), vascular invasion (p = 0.5879), ER status (p = 0.544), the presence of DCIS (p = 0.2966), lymph node status (p = 0.9412) or tumour stage (p = 0.0608–0.943). Table 2 shows the various p-values.

**Table 2:** Shows a summary of p-values of the associations between IGF-1 and IGF-1R mRNA and clinicopathological parameters

**Parameter**	**ER**	**DCIS**	**Grade**	**LN (1–3)**	**LN +/-**	**Age >60**	**Size >2 cm**	**Vasc Inv**
**IGF-1R**								
Tumour	0.5498	0.8456	0.8789	0.8832	0.9412	0.3683	0.855	0.5717
Normal	0.342	0.9643	0.7442	0.1691	0.2676	0.9382	0.8713	0.4742
T: N	0.1231	0.5249	0.6828	**0.0608**	0.2204	0.2733	0.2003	0.5073
**IGF-1**								
Tumour	0.544	0.2966	0.1084	**0.0657**	0.9384	0.115	0.7417	0.5879
Normal	0.8	0.1031	0.9513	**0.0032**	0.9144	0.1376	0.8699	0.4279
T: N	0.6857	0.107	0.1985	**0.0288**	0.1196	0.8906	0.8279	0.998

We observed an interesting significant association between IGF-1R and the type of surgical procedure performed (wide local excision/mastectomy) (p = 0.0288). Table 3 shows the raw data.

**Table 3:** Raw data

**Age**	**OP**	**Grade**	**Size mm**	**DCIS/grade**	**Vas.**	**LN**	**ER**	**IGF-1**	**IGF-1R**
53	WLE	3	25	No	No	0/8	Neg	0.610	0.166
78	WLE	1	22	Y/Low	No	1/15	Pos	0.724	0.189
88	Mast	3	110	No	Yes	Dif	Neg	0.719	0.067
90	WLE	3	23	No	No	0/13	Pos	1.037	0.036
83	Mast	3	70	No	Yes	17/17	Pos	0.854	0.177
58	Mast	3	90	No	Yes	6/13	Neg	0.403	0.169
54	Mast	2	32	Y/High	?	0/13	Pos	0.398	0.305
74	Mast	2	25	Y/High	No	1/12	Pos	0.489	0.235
85	Mast	2	14	No	NK	0/19	Neg	0.512	0.271
59	Mast	3	22	Y/High	No	1/5	Neg	0.499	0.180
72	Mast	3	35	Y/High	Yes	1/2	Neg	0.548	0.168
72	Mast	2	10	No	No	0/15	Pos	0.605	-
78	Mast	3	40	No	No	0/7	Neg	0.711	0.029
86	WLE	3	30	No	No	0/1	Pos	0.785	0.126
66	Mast	1	25	Y/High	No	0/11	Pos	0.186	0.076
77	WLE	3	30	Y/High	No	0/13	Neg	0.261	0.059
46	WLE	3	25	No	No	1/10	Neg	0.345	0.096
79	WLE	1	13	No	Yes	0/1	Pos	0.328	0.211
46	WLE	3	38	No	Yes	0/21	Neg	0.319	-
66	Mast	3	32	Y/NK	Yes	2/17	Pos	-	0.065
65	WLE	3	26	Y/High	Yes	1/20	Neg	0.562	0.129
78	Mast	2	22	No	No	0/12	Pos	0.546	0.352
45	WLE	2	20	No	No	1/28	Pos	0.426	0.159
37	WLE	3	20	Y/High	No	1/14	Neg	0.444	0.207
38	WLE	3	15	Y/High	No	1/7	Pos	0.370	0.122
41	WLE	3	23	No	Yes	6/7	Neg	0.158	0.074
74	WLE	3	36	No	No	0/13	Pos	0.187	0.106
70	WLE	2	35	Y/NK	Yes	5/18	Pos	0.178	0.114
62	WLE	2	26	Y/Inter	Yes	3/13	Pos	0.155	0.126
82	WLE	1	12	No	No	0/28	Pos	0.193	0.074
80	Mast	2	35	Y/Low	Yes	1/12	Pos	-	-

**Key: **WLE: Wide Local Excision

Mast: Mastectomy

Vas.: Vascular invasion

LN: Positive lymph node

NK: Not Known

Pos: Positive, Neg: Negative

Y: Yes

ER: Estrogen receptor status

Dif: Diffuse

Inter: Intermediate

## Discussion

Previous data shows that the interaction of insulin-like growth factors (IGFs) with the IGF-I receptor promotes cell proliferation, stimulates mitosis, combats apoptosis, and induces the transformation of normal cells to cancer cells [5,16-19].

IGFs seem to be freely available to the malignant epithelial cells from both endocrine and paracrine sources. However, the relative importance of endocrine production in normal breast tissues remains to be defined.

Earlier studies looked into the association between IGF-1/IGF-1R expression and clinicopathological features of breast cancer with conflicting results. Pappa V *et al *found no correlation between IGF1-R content and a variety of tumor parameters (tumor size, lymph node involvement, grade) and host characteristics (age, body mass index, menopausal status) [20]. Other studies, although looked at IGF-1 mRNA expression, failed to examine its relation to other clinical parameters [12,21,22].

The level of IGF-I mRNA expression seems to correlate with the nodal status, the best single prognostic indicator in human breast cancer. There is an increasing body of evidence that estrogen induces the expression of several members of IGF family including IGF-I, IGF-1R, IGF-II and IGF-2R [23-26]. The lack of correlation between ER status and both IGF-I and IGF-1R could be explained on the basis of the multiplicity of molecular markers influenced by estrogen.

The lack of correlation between both IGF-I and IGF-1R and patients' age, tumour size, grade, vascular invasion or the presence of DCIS are in consistence with the findings of Sancak *et al *[27] who found no association between the serum level of IGF-I and these clinicopathological parameters. It is important to note that protein expression does not always correlate with gene expression. The significance of the association between IGF-1R and the type of procedure performed is yet to be explained.

Our results should be interpreted with caution due to the small sample size.

The significant correlation we observed between nodal status and mRNA expression levels underscores the need for further studies with long-term clinical outcome data to validate the potential role of IGF-1 expression as a prognostic marker.
